# Effects of reward and punishment on the interaction between going and stopping in a selective stop-change task

**DOI:** 10.1007/s00426-016-0827-5

**Published:** 2016-11-25

**Authors:** Frederick Verbruggen, Rosamund McLaren

**Affiliations:** 1 School of Psychology, University of Exeter, Exeter, EX4 4QG UK; 2 Department of Experimental Psychology, Ghent University, 9000 Ghent, Belgium

## Abstract

Inhibition of no-longer relevant go responses supports flexible and goal-directed behavior. The present study explored if the interaction between going and stopping is influenced by monetary incentives. Subjects (*N* = 108) performed a selective stop–change task, which required them to stop and change a go response if a valid signal occurred, but to execute the planned go response if invalid signals or no signals occurred. There were two incentive groups: the punishment group lost points for unsuccessful valid-signal trials, whereas the reward group gained points for successful valid-signal trials. There was also a control group that could not win or lose points on any trials. We found that, compared with the control group, incentives encouraged subjects to slow down on no-signal trials, suggesting proactive control adjustments. Furthermore, latencies of valid change responses were shorter in the incentive groups than in the control group, suggesting improvements in executing an alternative response. However, incentives did not modulate stop latency or the interaction between going and stopping on valid-signal trials much. Finally, Bayesian analyses indicated that there was no difference between the reward and punishment groups. These findings are inconsistent with the idea that reward and punishment have distinct effects on stop performance.

**Electronic supplementary material:**

The online version of this article (doi:10.1007/s00426-016-0827-5) contains supplementary material, which is available to authorized users.

## Introduction

**Fig. 1:**
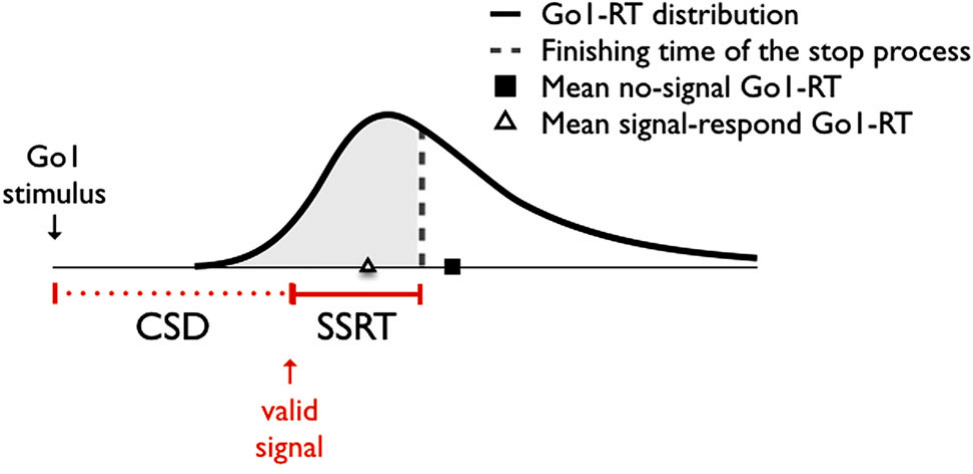
The independent race in a stop-change task. When the stop and go processes are independent, only the fastest responses escape inhibition (Logan & Cowan, 1984). Consequently, signal-respond Go1-RT should be shorter than no-signal Go1-RT: the former reflects the mean of the fastest responses that escaped inhibition (i.e. the responses on the *left* of the *vertical dotted line*), whereas the latter reflects the mean of the whole Go1-RT distribution. See Verbruggen and Logan (2015) for an elaborate discussion. *CSD* change-signal delay, *SSRT* stop-signal reaction time, which is the covert latency of the stop process

Response inhibition is a hallmark of executive control, and receives a great deal of attention across disciplines (Verbruggen & Logan, 2008a). Cognitive psychologists and neuroscientists have explored the cognitive and neural mechanisms of response inhibition, developmental scientists have studied the ‘rise and fall’ of inhibitory control capacities across the life span, and clinical researchers have examined correlations between individual differences in response inhibition and behaviors such as substance abuse, overeating, and risk-taking. A popular task to study response inhibition is the stop-signal task. In this task, subjects are instructed to respond quickly to a go stimulus (the go component of the task), but to withhold their response when a stop signal occurs after a variable stop-signal delay (the stop component of the task). On stop-signal trials, performance can be modeled as an independent race between a go process, triggered by the presentation of a go stimulus, and a stop process, triggered by the presentation of the stop signal (Logan & Cowan, 1984); go responses are successfully inhibited when the stop process finishes before the go process (signal-inhibit), but are incorrectly executed when the go process finishes before the stop process (signal-respond; Fig. 1). Thus, successful stop performance requires a ‘reactive’ system that quickly detects signals and activates the appropriate stop response. However, optimal performance in response-inhibition tasks also requires ‘proactive’ control to find a balance between competing task demands (i.e. responding quickly vs. stopping; Aron, 2011; Verbruggen & Logan, 2009a). In the present study, we examined how task balance and the race between going and stopping are influenced by monetary incentives in a selective stop-change task.

Many studies have shown that going and stopping are independent for most of their durations in standard stop-signal and stop-change tasks (e.g., Logan, 1981; Logan & Burkell, 1986; Verbruggen, Schneider, & Logan, 2008; Yamaguchi, Logan, & Bissett, 2012). For example, the independent horse-race model predicts that mean signal-respond RT should be shorter than mean no-signal RT, because the former only represents the mean of those responses that were fast enough to escape inhibition, whereas the latter represents the mean of all go responses (Fig. 1). This prediction has been confirmed by many stop-signal studies (Verbruggen & Logan, 2009b). Studies using stop-change tasks have provided further support for the independence assumption. In stop-change tasks, subjects are instructed to cancel the originally planned go response and execute an alternative ‘change’ response when a signal occurs. Experimental, computational, and neuro-imaging works suggest that subjects first inhibit the original go response and then execute the alternative change response; furthermore,  studies indicate that similar (neural) stopping mechanisms are involved in simple stop tasks and stop-change tasks (Boecker, Gauggel, & Drueke, 2013; Camalier et al., 2007; Elchlepp & Verbruggen, 2016; Jha et al., 2015; Verbruggen et al., 2008). Importantly, in stop-change tasks, stopping is also not influenced by go processing in the primary task (Logan & Burkell, 1986) or by the selection and execution of the change response (Verbruggen et al., 2008), which is consistent with the independent race model.

These stop-signal and stop-change findings are intriguing because most research on multitasking indicates that central-processing capacity^1^ is limited, resulting in a performance decrement when two stimuli associated with different tasks (or task components) are presented in rapid succession (Pashler, 1994). In other words, there is usually dependence when two or more tasks overlap. In standard stop-signal and stop-change tasks, stop and go processes do not seem to share capacity in this way (i.e. there is independence).

A different picture emerges when multiple stop signals are introduced. In selective stop-signal tasks, different signals are presented and subjects must stop if one of them occurs (valid signal), but not if the others occur (invalid signals). Thus, this task introduces a decisional component to the stop-signal task; as such, it may provide a richer model of action control than standard stop-signal or stop-change tasks. Bissett and Logan (2014) found that signal-respond RT was sometimes longer than no-signal RT in selective stop-signal tasks, suggesting that selecting the appropriate response to the signal interacts with ongoing go processes (violating the assumptions of the independence race model).^2^

The ‘dependence’ conclusion was further supported by a recent study that used a selective stop-change task to examine the interaction between going and stopping on signal trials (Verbruggen & Logan, 2015). In the primary task, subjects responded to a go stimulus (Go1 response). On some trials, a signal occurred. When the signal was valid, subjects had to stop the Go1 response and replace it with another response (Go2 or change response). When the signal was invalid, subjects had to execute the planned Go1 response (they had to ignore the signal). Signal validity was indicated by a cue at the beginning of a trial. For many subjects, latencies of Go1 responses on no-signal trials (no-signal Go1-RT) were shorter than Go1 latencies on valid-signal trials on which response inhibition failed (signal-respond Go1-RT) and Go1 latencies on invalid-signal trials (invalid-signal Go1-RT). This RT pattern was similar to the pattern observed in selective stop tasks in which subjects did not have to execute a secondary response (Bissett & Logan, 2014). However, these findings are inconsistent with the independent race model, which assumes that going and stopping are independent for most of their durations (Fig. 1; Logan & Cowan, 1984). Instead, they suggest that the decision to stop interfered with go processing. In other words, going and stopping are dependent and have to share limited central-processing capacity in selective stop tasks (Verbruggen & Logan, 2015).

The level of dependence (or interaction) between going and stopping may be influenced by response strategies. In this context, a strategy is defined as “an optional organization of cognitive resources or abilities that is designed to achieve some goal in some task environment” (Logan, 1985, p. 194). Several strategies can be used to perform a task, and which strategy is used at a particular moment can be influenced by voluntary decisions (e.g. subjects may determine their strategy at the beginning of a block; see e.g. Strayer & Kramer, 1994) and task-related or environmental factors (e.g. positive or negative outcomes, or the relative frequency of certain events). For example, Bissett & Logan (2014) found that signal-respond Go1-RT did not differ much from no-signal Go1-RT when most signals were invalid, but it was shorter when most signals were valid. This finding suggests that stopping was prioritized more when most signals were valid: When stopping is fully prioritized, the stop process is not influenced much by processing in the go task; hence, only the fastest trials can escape inhibition, as predicted by the independent race model (see Fig. 1). Research on dual-tasking provides further support for the idea that task prioritization can be influenced by strategic and environmental factors. When two stimuli are presented in rapid succession, prioritizing the first task leads to serial processing (i.e. central-processing in the Go2 task only starts when central-processing in the Go1 task is finished). This is often the most advantageous processing mode because it reduces response competition (Logan & Gordon, 2001; Meyer & Kieras, 1997). But in some situations, overall task performance may benefit from prioritizing both tasks more equally (Miller, Ulrich, & Rolke, 2009). For example, the likelihood of equal task prioritization (i.e. central-processing in the Go1 and Go2 tasks occurs simultaneously) increases when there are more short delays than long delays (Miller et al., 2009).

## The present study

In the present study, we examined if the balance or competition between going and stopping in selective stop tasks could be influenced by monetary incentives. Previous work indicates that incentive motivation can influence performance in standard stop-signal tasks (for a general review on motivation and cognition, see Braver et al., 2014). The influence of incentives on performance depends on how they are delivered or manipulated. In some studies, reward for successful stops was delivered in a block-based fashion (i.e. subjects were informed at the beginning of a block or run of trials that successful stop performance would be rewarded; see e.g. Greenhouse & Wessel, 2013; Leotti & Wager, 2010). This incentive manipulation enhanced stop performance on stop-signal trials, but slowed responding on no-signal trials. We observed similar findings in two pilot studies that are reported in Supplementary Materials:^3^ when successful stop/change was incentivized, go responses in the primary task were slower (despite a strict response deadline), but performance on signal trials was (numerically) improved. Combined, these studies indicate that subjects trade speed in the go task (e.g. by increasing response thresholds or adjusting attentional settings) for success in the stop task when successful stop performance is rewarded in a block-based (or experiment-based) fashion. Note that when go performance is rewarded, response latencies or accuracy on no-signal trials tend to decrease and stopping is impaired (Padmala & Pessoa, 2010). Thus, rewards can change the balance between going and stopping in both directions.

Incentives can influence stop performance in other ways as well. In a series of studies, Boehler and colleagues (e.g. Boehler, Hopf, Stoppel, & Krebs, 2012; Boehler, Schevernels, Hopf, Stoppel, & Krebs, 2014) showed reward-related information at the moment of the stop-signal presentation (i.e. the color of the stop signal indicated whether subjects would receive an extra reward for successful stop performance or not). They found that SSRT was shorter and that key regions of the neural inhibitory control network were activated more on reward trials than on non-reward trials (for a review, see Krebs, Hopf, & Boehler, 2016). These findings cannot be attributed to a simple trade-off between going quickly and stopping, because the reward signal is presented after the presentation of the go stimulus. Of course, global attentional and response settings could be influenced by the occasional delivery of reward; thus, even in the studies of Boehler and colleagues, proactive control or task settings could be modulated by reward (Schevernels et al., 2015). Furthermore, a study by Rosell-Negre et al. (2014) indicates that incentives can influence strategy adjustments after signal trials. In sum, previous studies indicate that performance on stop-signal trials in standard stop tasks (i.e. with only one signal) improves when incentives for successful stopping are provided, which could be due to preactivation of the stopping network, control adjustments, or both.

In the present study, we examined if incentives could change the balance between going and stopping and the degree of dependence or capacity sharing in selective stop situations. We explained the incentive structure at the beginning of the experiment and it remained the same throughout the whole experiment. Furthermore, we incentivized stopping only. Based on previous studies, we predicted that this incentive scheme would encourage subjects to make proactive strategy adjustments at the beginning of the task (cf. Strayer & Kramer, 1994). Such adjustments could influence responding on no-signal trials as subjects trade speed in the go task for success in the stop task (see above). Furthermore, we predicted that incentives would influence the interaction between going and stopping on valid-signal trials: when stopping is prioritized (due to the incentives for stopping), it will not be influenced much by going (i.e. independence); by contrast, when go and stop processing are prioritized more equally on signal trials, stopping will be influenced by ongoing go processes (i.e. dependence).

We included two incentive conditions, namely a reward condition and a punishment condition. Previous work suggests that reward and punishment can have distinct effects on go and stop performance (Guitart-Masip et al., 2012; Verbruggen, Best, Bowditch, Stevens, & McLaren, 2014). Furthermore, reward and punishment schemes may influence strategy selection differently. For example, Braver, Paxton, Locke, and Barch (2009) found that a reward scheme encouraged a proactive control mode, whereas a punishment scheme encouraged a more reactive control mode.

Even though we were mostly interested in strategy selection (i.e. how a task is performed) and the balance between going and stopping, we also wanted to explore the effects of incentives on reactive control measures (as previous studies, as mentioned above, found effects of incentives on both proactive and reactive control). Therefore, we used a selective stop-change task (instead of a selective stop task), because it provides us with two measures of ‘reactive’ action control on valid-signal trials: the latency of the stop response (stop-signal reaction time or SSRT) and the latency of the change response (see also Verbruggen & Logan, 2015). As noted above, the underlying response-inhibition mechanisms in stop and stop-change tasks are very similar. However, SSRT can only be estimated when the assumptions of the race model are met, whereas the latency of the change response is measured directly. In other words, the stop-change task provides an index of reactive action control even when the assumptions of the independence race model are violated (and we expected such violations, especially in the control condition).

## Experiment

**Fig. 2:**
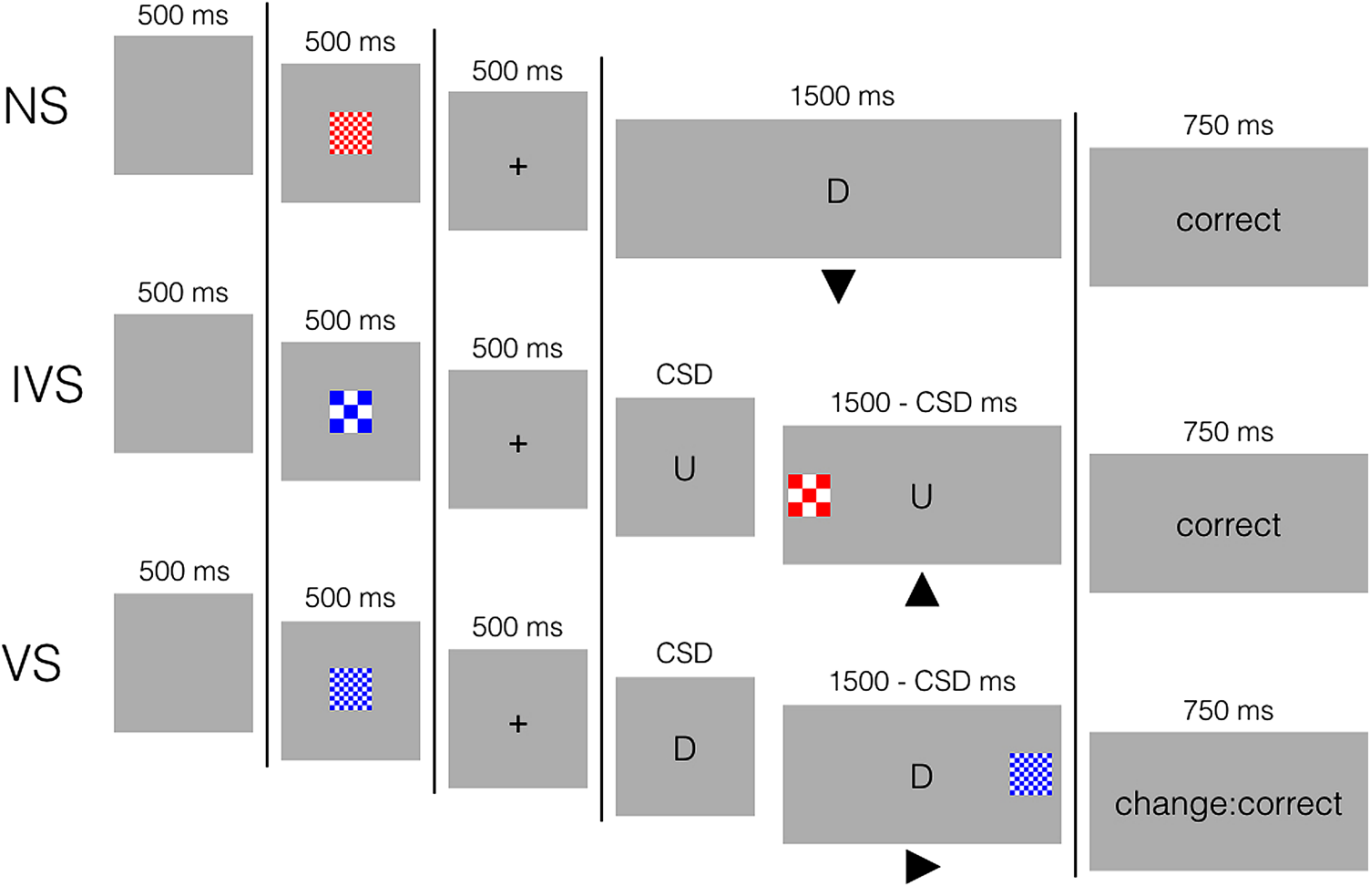
Examples of the three trial types in the selective stop-change task. The *top panel* shows the sequence of events on no-signal trials (NS). The *middle panel* shows the sequence of events on invalid-signal trials (IVS), and the *bottom panel* shows the sequence of events on valid-signal trials (VS). Signal validity was indicated by the cue (the centrally presented chequerboard) at the beginning of the trial. The *arrows under* the *letters* indicate the correct response. *CSD* change-signal delay. See the “Method” section for further details

In the primary task, subjects responded to a letter (Go1 response). On some trials, a signal appeared on the left or right of the go stimulus (Fig. 2). When the signal was valid, subjects had to stop their planned Go1 response and respond to the location of the signal instead (Go2 or change response). When the signal was invalid, subjects had to ignore it and execute the planned Go1 response. Signal validity was indicated by a visual cue at the beginning of a trial (Fig. 2). There were three groups. The punishment group lost points for unsuccessful valid-signal trials. The reward group gained points for successful valid-signal trials. Finally, the control group could not win or lose points on any trials.

Incentives may encourage subjects to make proactive strategy adjustments (see above). Such adjustments often influence responding on no-signal trials as subjects trade speed in the go task for success in the stop task (Aron, 2011; Verbruggen & Logan, 2009a). Therefore, in a first analysis, we examined how Go1-RTs on no-signal trials changed over time in the three groups. We predicted that incentives on valid-signal trials would encourage subjects to slow down (i.e. alter their speed/accuracy trade-off). Note that we focused on Go1-RTs only to get a ‘pure’ measure of proactive control adjustments; after all, stop-change performance on successful valid-signal trials is influenced by both proactive and reactive control processes. We used a similar analysis approach in our previous studies that examined proactive inhibitory control (e.g. Elchlepp, Lavric, Chambers, & Verbruggen, 2016; Verbruggen & Logan, 2009a).

In a second analysis, we compared Go1-RTs on no-signal and failed valid-signal trials to examine the interaction between going and stopping on signal trials. We predicted that the ‘no-signal Go1-RT minus signal-respond Go1-RT’ difference would be larger in the incentive conditions than in the control condition. When stopping is prioritized on valid-signal trials, stopping is not influenced much by going; consequently, signal-respond Go1-RT should be shorter than no-signal Go1-RT (Bissett & Logan, 2014; Logan & Cowan, 1984; Fig. 1). By contrast, when go and stop processing are prioritized more equally on signal trials, stopping is influenced by ongoing go processes; consequently, the difference between signal-respond and no-signal Go1-RT should become smaller or even reverse (Bissett & Logan, 2014; Verbruggen & Logan, 2015). For completeness, we also analyzed invalid-signal Go1-RT.

In a third analysis, we analyzed change (Go2) performance and explored the correlation between response-slowing and improvements in change performance. We also analyzed stop-signal latencies of subjects for which the assumptions of the independent race model were not violated. Finally, we report an exploratory analysis of sequential effects in the three conditions.

### Method

#### Subjects

108 volunteers (36 per condition) from the University of Exeter participated for monetary compensation (£5) or partial course credit. The number of subjects was determined in advance, based on a power calculation for the main effects of interest. As indicated above, effects of reward on strategy selection and task prioritization could be tested by comparing RTs for the different trial types. In our previous study, the RTs correlated strongly (e.g. the correlation between no-signal Go1-RT and invalid-signal Go1-RT was *r*(191) = 0.93, *p* < 0.001; Verbruggen & Logan, 2015). Therefore, a power calculation indicated that the present experiment was sufficiently powered (0.80) to detect between–within factor interactions with a small effect size. Note that for completeness, we also analyzed change-RTs and SSRTs in the three conditions. However, we could only detect (very) large effects in these analyses (with power = 0.80); so, these ‘reactive’ control results should be interpreted with caution.

*p*(correct) on valid-signal trials was close to 0.50 for most subjects in Verbruggen and Logan (2015). Therefore, we had decided (before data collection had started) to replace subjects for which *p*(correct) >0.70 or *p*(correct) <0.30 in the present study. Three subjects (control: 1; punishment: 2) were replaced. We used the integration method to estimate SSRT (see below); therefore, we used a more lenient exclusion criterion than the one used for the mean SSRT estimation method (e.g. Verbruggen, Logan, & Stevens, 2008).

#### Apparatus, stimuli and procedure

The experiment was run on a 21.5-inch iMac using Psychtoolbox (Brainard, 1997). The Go1 stimuli were the letters ‘U’ and ‘D’ (size: approximately 2 × 4 mm). Subjects responded to them by pressing the ‘up’ (U) and ‘down’ (D) arrow keys of a standard keyboard with their right middle finger. The Go1 stimuli were centrally presented in a black font (Courier) on a light grey background (RGB = 175 175 175). There were four stop-change signals (chequerboards; size: 12 × 12 mm), which varied along two dimensions: the number of squares inside the board (3 × 3 or 9 × 9), and the color (red: RGB = 255 0 0, or blue: RGB = 0 0 255). Signals appeared approximately 4 cm on the left or right of the Go1 stimulus. Subjects responded to the location of valid signals (Go2 or change response) by pressing the corresponding arrow key with their right index (left arrow) or right ring (right arrow) finger.

All trials started with the presentation of a signal cue (one of the chequerboards) in the center of the screen for 500 ms (Fig. 2). This cue indicated the valid signal, which could change on every trial. The cue was replaced by a black fixation cross for 500 ms, after which a letter (the Go1 stimulus) appeared. Subjects had to decide whether the letter was ‘U’ or ‘D’. The letter remained on the screen for 1500 ms, regardless of RT (a similar maximum RT has been used in previous stop-signal studies).

On 1/3 of the trials, a signal was presented on the left or right of the letter after a variable delay. When the signal matched the cue (valid signal), subjects had to withhold the Go1 (up/down) response and respond to the location of the signal instead (Go2 response; left/right). When the signal did not match the cue (invalid signal), subjects had to ignore it and execute the planned Go1 response. Consistent with our previous research (Verbruggen & Logan, 2015), the location of the signals was randomized and the four signals occurred with equal probability in random order. Thus, only 25% of the signal trials—or 8.33% of all trials—were valid-signal trials, and trial types were fully randomized. The change-signal delay (CSD) was initially set at 250 ms and continuously adjusted according to a tracking procedure to obtain a probability of successful valid-change performance of 0.50. Each time a subject responded to the Go1 stimulus or failed to execute the correct Go2 response on a valid-signal trial, CSD decreased by 50 ms. When subjects successfully replaced the Go1 response on a valid-signal trial, CSD increased by 50 ms. Subjects were informed about this tracking procedure and they were told not to wait for a change signal to occur. CSD for invalid-signal trials was yoked to the valid-signal CSD.

At the end of each trial, we presented feedback for 750 ms. On no-signal and invalid-signal trials, we presented ‘correct’, ‘incorrect’, or ‘too slow’ (in case subjects did not respond before the end of the trial). The feedback message on valid-signal trials differed between groups. In the punishment group, we presented: ‘change: correct’ when subjects successfully replaced the Go1 response, or ‘change: incorrect. You lose 40 points’ when subjects executed the Go1 (up/down) response or executed an incorrect Go2 (left/right) response. In the reward group, we presented ‘change: correct. You win 40 points’ for successful valid-signal trials, or ‘change: incorrect’ for unsuccessful valid-signal trials. In the control group, we presented ‘change: correct’ or ‘change: incorrect’ for successful and unsuccessful valid-signal trials, respectively. The next trial started after a further 500 ms.

Subjects in the punishment and reward groups were informed at the beginning of the experiment that the points would be converted into money (100 points = £0.1) at the end of the experiment, but only if overall performance on no-signal and invalid-signal trials was also satisfactory (i.e. if they responded correctly and in time on the majority of trials). The start balance was 2500 points in the Punishment group, and 0 points in the Reward group. There were 64 valid-signal trials in the experiment. Due to the tracking procedure, both groups ended with approximately 1250 points (£1.25).

The experiment consisted of 768 trials in total. Subjects received a break after every 64 trials. During the break, we presented subjects’ mean no-signal Go1-RT, the number of incorrect and missed no-signal responses, and the percentage of correctly replaced responses on valid-signal trials. Subjects had to pause for 15 s.

### Analyses

All data processing and analyses were completed using R. All data files and R scripts are deposited on the Open Research Exeter data repository (http://hdl.handle.net/10871/24540).

**Fig. 3:**
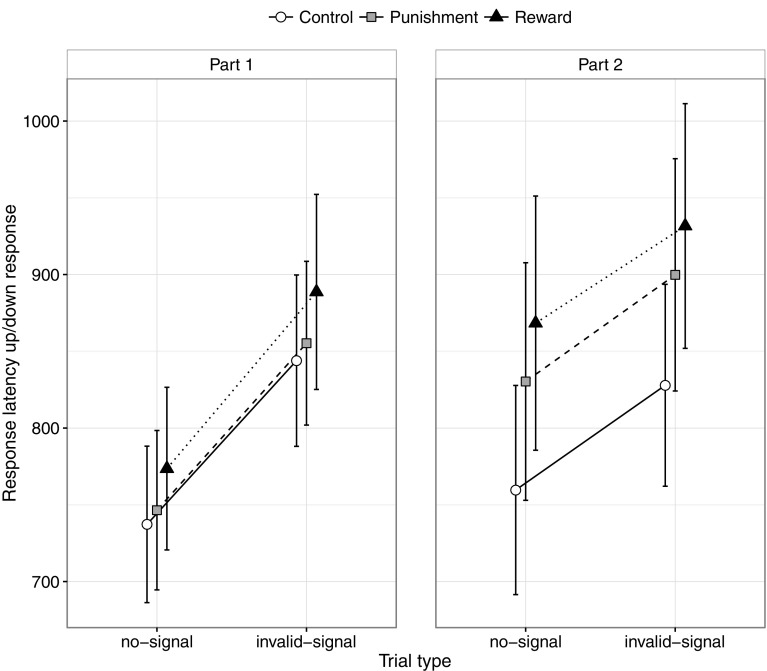
Latencies of correct Go1 responses as a function of part (first half or second half of the experiment), group (control, punishment, and reward), and trial type (no-signal vs. invalid-signal). *Error bars* indicate 95% confidence intervals

Descriptive and inferential statistics appear in Tables 1, 2, 3, 4, 5 and Fig. 3. We also calculated Bayes factors for all main effects and interaction contrasts in the ANOVA designs, and present an overview of these analyses in Supplementary Materials. Part (first half vs. second half of the experiment) was included in the analyses, because go performance may gradually change over time in the incentive conditions (Leotti & Wager, 2010). Furthermore, reward and punishment can influence learning in response-inhibition tasks (Guitart-Masip et al., 2012; but see also Krebs et al. (2016), for a discussion of reward and practice effects).

**Table 1:** Overview of the analyses of variance

Analysis	*df*1	*df*2	SS1	SS2	*F*	*p*	*η*_gen_^2^
No-signal Go1-RT							
Group	2	105	190,054	7,118,477	1.402	0.251	0.024
Part	1	105	242,421	704,151	36.149	**<0.001**	0.030
Group by part	2	105	54,819	704,151	4.087	**0.020**	0.007
Signal-respond vs. no-signal Go1-RT							
Group	2	105	347,529	12,729,865	1.433	0.243	0.024
Part	1	105	333,233	1,180,176	29.648	**<0.001**	0.023
Trial type	1	105	183,236	297,773	64.612	**<0.001**	0.013
Group by part	2	105	79,230	1,180,176	3.525	**0.033**	0.005
Group by trial type	2	105	1327	297,773	0.234	0.792	0.000
Part by trial type	1	105	12,088	175,807	7.219	**0.008**	0.001
Group: part: trial type	2	105	2851	175,807	0.851	0.430	0.000
Invalid-signal vs. no-signal Go1-RT							
Group	2	105	389,550	14,400,830	1.420	0.246	0.024
Part	1	105	222,712	1,237,161	18.902	**<0.001**	0.014
Trial type	1	105	847,767	176,208	505.174	**<0.001**	0.051
Group by part	2	105	96,683	1,237,161	4.103	**0.019**	0.006
Group by trial type	2	105	70	176,208	0.021	0.979	0.000
Part by trial type	1	105	50,348	80,366	65.780	**<0.001**	0.003
Group: part: trial type	2	105	1011	80,366	0.661	0.519	0.000
Change-RT							
Group	2	105	264,002	2,094,082	6.619	**0.002**	0.099
Part	1	105	257,662	315,288	85.809	**<0.001**	0.097
Group by part	2	105	3886	315,288	0.647	0.526	0.002
Go1-RT difference (sequential analysis)							
Group	2	105	3025	244,491	0.650	0.524	0.004
Properties previous trial	3	315	194,837	575,693	35.536	**<0.001**	0.192
Group by previous trial	6	315	14,623	575,693	1.333	0.242	0.018

Latencies were analyzed by means of mixed ANOVAs with group (control, punishment, reward) as a between-subjects factor, and part (first half. vs. second half of the experiment) as within-subjects factor. For the ‘invalid-signal vs. no-signal’ and ‘signal-respond vs. no-signal’ analyses, we also included trial type as a within-subjects factor. For the sequential analysis, we analyzed the Go1-RT difference between trials as a function of the properties of the previous trial (correct no-signal, correct invalid-signal, unsuccessful valid-signal, or successful valid-signal trial). *p*’s < 0.05 are in bold

**Table 2:** Overview of planned comparisons to explore the Group by Part interaction for the latencies in the primary task (first and second set of comparisons), the main effect of group for latencies of the change response and stop response on valid-signal trials (the third set and fourth of comparisons), and the main effect of ‘previous trial properties’ in the sequential analysis (fifth set of comparisons)

Comparison	Diff	Lower CI	Upper CI	*df*	*t*	*p*	BF	*g*
No-signal Go1-RT: within-group differences								
Control: part 1 vs. part 2	−22	−58	13	35	−1.267	0.214	0.374	0.126
Punish: part 1 vs. part 2	−84	−119	−48	35	−4.788	**0.001**	739.642	0.434
Reward: part 1 vs. part 2	−95	−140	−49	35	−4.244	**0.001**	169.169	0.467
No-signal Go1-RT: between-group differences								
P1: control vs. punish	−9	−81	62	70	−0.258	0.797	0.25	0.06
P1: control vs. reward	−36	−109	36	70	−1.003	0.319	0.374	0.234
P1: punish vs. reward	−27	−100	46	70	−0.741	0.461	0.308	0.173
P2: control vs. punish	−71	−172	31	70	−1.392	0.168	0.556	0.325
P2: control vs. reward	−109	−214	−3	70	−2.059	0.043	1.462	0.48
P2: punish vs. reward	−38	−149	73	70	−0.681	0.498	0.297	0.159
Change-RT: between-group differences								
Control vs. punish	67	18	117	70	2.73	**0.008**	5.458	0.636
Control vs. reward	79	31	128	70	3.271	**0.002**	19.885	0.763
Punish vs. reward	12	−31	55	70	0.561	0.576	0.278	0.131
SSRT: between-group differences								
Control vs. punish	19	−19	58	37	1.023	0.313	0.471	0.321
Control vs. reward	31	−3	65	40	1.806	0.078	1.093	0.549
Punish vs. reward	11	−22	44	41	0.678	0.502	0.362	0.203
No-signal RT difference: property of previous trial								
No-signal vs. invalid	−39	−45	−33	107	−13.191	**<0.001**	1.14 × 10^21^	2.300
No-signal vs. signal-respond	−57	−70	−44	107	−8.641	**<0.001**	1.19 ×10^11^	1.553
No-signal vs. signal-inhibit	−45	−57	−32	107	−7.210	**<0.001**	1.08 ×10^8^	1.294
Invalid vs. signal-respond	−17	−29	−5	107	−2.765	**0.007**	3.916	0.403
Invalid vs. signal-inhibit	−5	−17	7	107	−0.861	0.391	0.153	0.129
Signal-respond vs. signal-inhibit	12	0	24	107	1.925	0.057	0.630	0.199

*p*’s < 0.05 after Holm–Bonferroni correction for multiple comparisons are in bold

The Bayes factor (BF) is an odds ratio: it is the probability of the data under one hypothesis relative to that under another. Evidence categories for Bayes factor: BF < 0.33 = substantial evidence for H_0_; 1/3 − 1 = anecdotal evidence for H_0_; 1 = no evidence; 1–3 = anecdotal evidence for H_A_; 3 − 10 = substantial evidence for H_A_; BF > 10 = strong to decisive evidence for H_A_. H_0_ = no difference between the trial types; H_A_ = a difference between the trial types. We calculated the Bayes factors with the Bayes factor package in R, using the default prior (0.707). For the SSRT analysis, we excluded subjects whose signal-respond RT was longer than their no-signal RT

**Table 3:** Overview of performance on valid-signal trials: probability of responding on a valid-signal trial [*p*(respond)], average valid change-signal delay (CSD), average reaction time for Go1 responses on signal-respond trials (signal-respond Go1-RT), the difference between signal-respond Go1-RT and no-signal Go1-RT (both correct and incorrect responses were included when mean no-signal RT was calculated), and average reaction time for correct Go2 responses (change-RT), as a function of part (first vs. second half of the experiment) and group (control, punishment, reward)

Independent variables	*p*(respond)		CSD		Signal-respond Go1-RT		No-signal Go1-RT minus signal-respond Go1-RT		Change-RT	
	*M*	SD	*M*	SD	*M*	SD	*M*	SD	*M*	SD
Part 1										
Control	0.386	0.102	380	135	703	136	35	69	693	131
Punish	0.402	0.097	388	141	723	152	23	75	631	99
Reward	0.367	0.124	431	149	742	177	34	76	624	99
Part 2										
Control	0.496	0.100	449	234	717	171	42	57	634	113
Punish	0.469	0.070	544	265	776	200	54	62	562	101
Reward	0.452	0.072	606	273	809	212	59	63	544	96

Change-RT corresponds to the time interval between the presentation of the valid signal and the left/right key press. Mean probability of not executing any response on valid-signal trials was 0.02 (SD = 0.13)

**Table 4:** Overview of the number of subjects and stop performance on valid-signal trials after exclusion of subjects whose signal-respond RT was longer than their no-signal Go1-RT (see “Analyses” section for further details): probability of responding on a valid-signal trial [*p*(respond)], average valid change-signal delay (CSD), stop-signal reaction time (SSRT)

Group	*N*	*p*(respond)		CSD		SSRT	
		*M*	SD	*M*	SD	*M*	SD
Control	19	0.41	0.11	486	206	269	76
Punish	20	0.40	0.08	568	201	249	69
Reward	23	0.39	0.10	597	212	238	59

For this subset of subjects, mean probability of not executing any response on valid-signal trials was 0.04 (SD = 0.05)

**Table 5:** No-signal RT difference as a function of the previous trial and group

Group	No-signal		Invalid-signal		Signal-respond (unsuccessful valid)		Signal-inhibit (successful valid)	
	*M*	SD	*M*	SD	*M*	SD	*M*	SD
Control	−13	12	22	25	57	67	27	57
Punish	−14	10	30	21	43	52	40	50
Reward	−12	11	27	24	30	64	28	66

For the reasons discussed above, we focused primarily on Go1-RTs in the analyses reported below. For completeness, we analyzed latency of the stop response (SSRT) and the change response on successful valid-signal trials as performance on these trials could be influenced by changes in reactive control, proactive control, or both. We calculated SSRT using the integration method (Verbruggen, Chambers, & Logan, 2013). To account for response-slowing, we calculated SSRT for each part separately, and then took the average (as recommended in Verbruggen et al., 2013). The independent race model assumes that stopping and going are independent for most of their durations. This assumption should not be taken lightly, because SSRT cannot be reliably estimated when it is violated. Therefore, we compared signal-respond Go1-RT with no-signal Go1-RT for each subject and part, and excluded subjects when signal-respond Go1-RT was longer than no-signal Go1-RT in part 1, part 2, or both. We had to exclude 46 subjects in total. The number of subjects per group appears in Table 4.

We also performed an exploratory sequential analysis in which we compared no-signal performance on trials that followed a correct no-signal trial, a correct invalid-signal trial, an unsuccessful (signal-respond) valid-signal trial, or a successful (signal-inhibit) valid-signal trial. There were not enough incorrect no-signal and invalid-signal trials to explore how Go1 errors influenced subsequent performance. For similar reasons, we could not explore how sequential effects influenced performance on invalid- and valid-signal trials. Measurements of post-signal slowing can be contaminated by global fluctuations in performance over the course of an experiment (Nelson, Boucher, Logan, Palmeri, & Schall, 2010). For example, when RTs gradually become longer in a block, probability of stopping will temporarily decrease (as the tracking procedure may need some time to catch up). This will also influence the measurement of post-signal slowing, because trials that follow a successful stop are more likely to come from slower parts of the block or experiment than trials that follow an unsuccessful stop. There is a solution for this problem: post-signal slowing can be quantified as the RT difference between the post-signal trial and the last preceding no-signal trial (Nelson et al., 2010; see Dutilh et al., 2012 for a similar solution to control for global fluctuations in post-error paradigms). For example, when a no-signal trial (trial *n*) was preceded by another no-signal trial (trial *n* − 1), the RT difference is ‘RT trial *n*’ minus ‘RT trial *n* *−* 1’. If trial *n*-*1* was an invalid-signal trial but trial *n* − 2 was a no-signal trial, the RT difference is ‘RT trial *n*’ minus ‘RT trial *n* – 2’.

Finally, we report the descriptive and inferential statistics for the accuracy data of the go task in “Appendix 1”. The accuracy data for the change task appear in Table 3. Note that we used a tracking procedure to determine the change-signal delay (like most stop-signal and stop-change studies; see Verbruggen et al., 2013). This procedure typically results in a p(respond|signal) ≈ 0.50, and compensates for individual or group differences in go or stop latencies. Therefore, incentives were not expected to influence the probability of executing the primary-task response on valid-signal trials. However, they could influence the latency of the change response and SSRT.

### Results

#### No-signal Go1-RT

No-signal Go1-RT increased substantially from the first half to the second half of the experiment in the punishment group (part 1: *M* = 746 ms; part 2: *M* = 830 ms; difference: *p* < 0.001, BF = 739) and reward group (part 1: *M* = 774 ms; part 2: *M* = 868 ms; difference: *p* < 0.001, BF = 169), but not in the control group (part 1: *M* = 737 ms; part 2: *M* = 760 ms; difference: *p* = 0.214, BF = 0.374). The group by part interaction was significant, *p* = 0.020 (Table 1). None of the other between-group differences was statistically significant after correction for multiple comparisons (Table 2).

No-signal RTs were generally long (considering the simplicity of the primary up/down task). This suggests that dual-task demands (i.e. updating and maintaining the relevant signal rule in working memory and monitoring for the signal) and response-strategy adjustments influenced performance in all groups, including the control group (Verbruggen & Logan, 2009a). However, the group by part interaction indicates that incentives encouraged subjects to slow down even more throughout the experiment.

#### Signal-respond vs. no-signal Go1-RTs

The independent race model assumes independence between going and stopping; so, mean signal-respond RT (i.e. RTs for trials on which a valid signal was presented but subjects executed the up/down Go1 response instead of the left/right Go2 response) should be shorter than mean no-signal RT (see Fig. 1). The model does not make any further assumptions about whether the executed response should ‘match’ the stimulus (i.e. up for ‘U’ and down for ‘D’) or not. Therefore, we included all executed Go1 responses for this analysis (including trials when subjects pressed ‘up’ for D and down for ‘U’; see also Verbruggen & Logan, 2015). Note that we have repeated the analysis after exclusion of non-matching responses, but this did not alter the main findings (see Supplementary Materials).

Descriptive statistics appear in Table 3. Consistent with the independent race model, signal-respond Go1-RT was on average 41 ms shorter than no-signal Go1-RT (main effect of trial type: *p* < 0.001). However, Figure S1 in Supplementary Materials shows that the independence assumption was violated for approximately 25–30% of the subjects in each group. In other words, for these subjects, we observed dependence or competition between going and stopping. This is consistent with our previous research (Verbruggen & Logan, 2015) and the findings of Bissett and Logan (2014). Importantly, the Go1-RT difference was similar in the three groups (control: 39 ms, punishment: 39 ms, reward: 46 ms; interaction group by trial type: *p* = 0.792). This conclusion was further supported by the Bayesian analyses (Supplementary Materials). Thus, incentives did not influence the dependence between going and stopping (or task prioritization) on valid-signal trials. After all, the difference between signal-respond RTs and no-signal RTs should have been larger when stopping was prioritized more.

The significant interaction between part and trial type (*p* = 0.008; Table 1) indicates that the signal-respond/no-signal difference increased throughout the experiment (part 1 = 31 ms; part 2 = 52 ms). The group by part interaction (*p* = 0.033) was the only significant group-related effect, and provides further support for the idea that RTs generally increased throughout the experiment in the incentive conditions.

#### Invalid-signal vs. no-signal Go1-RTs

Go1-RTs were generally longer on invalid-signal trials (875 ms) than on no-signal trials (786 ms), which is consistent with previous research (Bissett & Logan, 2014; Verbruggen & Logan, 2015). The significant interaction between trial type and part (*p* < 0.001; Table 2) indicates that this difference decreased throughout the experiment (part 1: 110 ms; part 2: 67 ms). Importantly, the Go1-RT difference was similar in the three groups (control: 87 ms; punishment: 89 ms; reward: 89 ms; group by trial type interaction: *p* = 0.979), and was observed for all subjects (Figure S1). The outcomes of the Bayesian analysis and the ANOVA were consistent. Thus, the ‘invalid-signal vs. no-signal’ comparison indicates that incentives did not influence how subjects processed invalid signals. The corresponding RT distributions (see Supplementary Materials) further supported this conclusion.

#### Performance on valid-signal trials

Change-RT (the latency of correct Go2 responses) was measured directly, so violations of the independence assumption (Verbruggen & Logan, 2015) and strategic slowing (Verbruggen et al., 2013) were not a concern. As can be seen in Tables 2 and 3, change-RTs were longer in the control group than in the punishment (difference = 67 ms; *p* = 0.008, BF = 5.458) and reward (difference = 79 ms; *p* = 0.002, BF = 19.885) groups. There was no difference between the incentive conditions (*p* = 0.576; BF = 0.278). Thus, incentives reduced the latency of change responses. Change-RT decreased with practice, but the Group by Part analysis was not significant (Table 1), indicating that incentives did not enhance practice effects (for a similar finding in a simple stop task, see Boehler et al., 2014; see also Krebs et al., 2016).

**Fig. 4:**
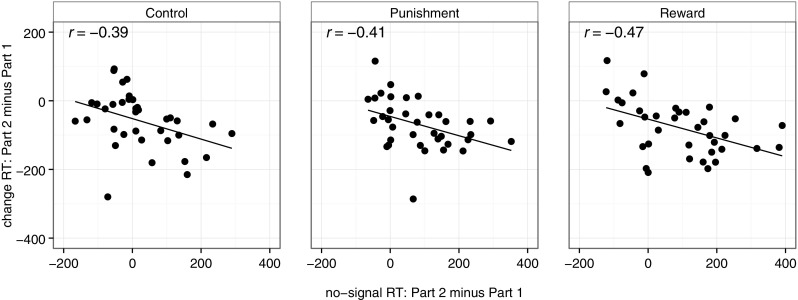
Correlation between the response-slowing on no-signal trials (no-signal Go1-RT: part 2 minus part 1) and improvements in change-RT on valid-signal trials (change-RT: part 2 minus part 1). A negative correlation indicates that subjects who slow more over time show greater improvements in change-RT

The no-signal RT analyses indicate that incentives encouraged subjects to slow down the primary-task response throughout the experiment (i.e. they made extra proactive control adjustments). We tested whether these adjustments influenced change-RTs. We correlated response-slowing in the primary go task (i.e. no-signal RT part 2 minus no-signal RT part 1) with stop-change performance (i.e. change-RT part 2 minus change-RT part 1). We found a negative correlation: when Go1-RT increased throughout the experiment, change-RT decreased, *r*(107) = −0.43, *p* < 0.001. Interestingly, this negative correlation was observed in each group (Fig. 4). Thus, proactive control adjustments influenced performance on valid-signal trials, even when no extrinsic incentives were provided.

As can be seen in Table 4, there were small numerical SSRT differences between the groups. However, these differences were not statistically significant, and the Bayes factors were inconclusive (Table 2). It could be argued that no SSRT differences were observed because the sample size was further reduced compared with the change-RT analyses. Therefore, we also analyzed change-RT after exclusion of those subjects for which the independence assumption of the race model was violated. There were still large change-RT differences between the incentive groups and the control group (“Appendix 2”). In other words, the change-RT pattern was not influenced much by the exclusion of subjects whose signal-respond RT was longer than their no-signal RT.

We found no reliable effects of reward and punishment on SSRT in this experiment. In pilot experiment 2 (see Supplementary Materials), in which we used a stop-signal task with only one signal, we also found no reliable effects of reward and punishment on SSRT after correction for multiple comparisons, and the Bayesian analyses were inconclusive. However, we observed some numerical trends that were consistent with the trends observed here and differences observed in previous studies (e.g. Boehler et al., 2012; Greenhouse & Wessel, 2013). Therefore, we combined the results of the main experiment and the pilot experiment by calculating meta-analytic Bayes factors for multiple *t* tests (Rouder & Morey, 2011). This meta-analysis revealed that reward had some beneficial effect on SSRT (BF_meta_ for reward vs. control = 5.11). The punishment vs. control comparison was still inconclusive (BF_meta_ = 0.65), whereas the reward vs. punishment comparison provided substantial support for the null hypothesis (BF_meta_ = 0.14). In sum, we can conclude that the incentives (and reward in particular) can have a beneficial effect on stop latencies, but large sample sizes are required to detect these differences. Because SSRT has to be estimated, it may be a noisier measure than go latencies, which can be measured directly.

#### Sequential effect of signal presentation

The results of the experiment suggest that reward and punishment influenced the response strategies. In a final exploratory analysis, we tested if incentives also influenced post-change-signal performance. In standard stop-signal tasks, response latencies are often slower after stop-signal trials than after no-stop-signal trials (Bissett & Logan, 2011, 2012; Nelson et al., 2010; Rieger & Gauggel, 1999; Verbruggen, Logan, Liefooghe, & Vandierendonck, 2008; see also Verbruggen & Logan, 2008c, for a similar observation in a stop-change task with only one signal). Bissett and Logan (2011) contrasted several accounts of post-stop-signal slowing, and found most support for a strategic adjustment account that proposes that stop-signal presentation encourages subjects to shift priority from the go task to the stop task. Such a shift produces longer response latencies after a signal trial and can reduce SSRT when the stop-signal modality remains the same (Bissett & Logan, 2012; for similar improvements in stop latencies in continuous variants of the stop-signal task, see Morein-Zamir, Chua, Franks, Nagelkerke, & Kingstone, 2007; Verbruggen & McLaren, 2016). Findings of Rosell-Negre et al. (2014) indicate that incentives can influence strategy adjustments after signal trials. Therefore, we also compared no-signal performance on trials that followed a correct no-signal trial, a correct invalid-signal trial, an unsuccessful (signal-respond) valid-signal, or a successful (signal-inhibit) valid-signal trial. As discussed in the “Analyses” section, post-change-signal slowing was quantified as the RT difference between the post-signal trial and the last preceding no-signal trial. Positive scores indicate that subjects are slower than on the previous no-signal trial; negative scores indicate that they were faster.

The descriptive statistics appear in Table 5. A univariate analysis revealed that properties of the previous trial influenced no-signal RT, but there was no main effect of group (control, reward, or punishment) or a group by trial type interaction (Table 1). In other words, incentives did not modulate sequential effects in our study. This conclusion was further supported by Bayesian ANOVA (see Supplementary Materials). To explore the main effect of trial type in more detail, we performed a series of post hoc tests. These appear in Table 2. As can be seen, no-signal RTs were generally longer after both valid- and invalid-signal trials than after no-signal trials (see also Table 5). There was no difference between trials that followed invalid-signal trials, unsuccessful valid-signal trials, or valid-signal trials. In other words, stop-signal presentation generally slowed responding on the subsequent trial, which seems consistent with the strategic adjustment account of Bissett and Logan (2011). Note that previous studies have also shown that the slowing is more pronounced when features of the previous trial are repeated (e.g. Verbruggen et al., 2008); unfortunately, we could not test this here because the number of valid-signal trials was too low.

## General discussion

### Incentives induce general slowing but do not influence the competition between going and stopping on signal trials

No-signal Go1-RTs increased more throughout the experiment in the reward and punishment groups than in the control group. The slowing in both groups can be attributed to extra ‘proactive inhibitory control’ adjustments. When subjects expect a stop signal, they monitor the environment and selectively attend to stop-signal features (e.g. Elchlepp et al. 2016), and downregulate attentional resources in the go task (e.g. Langford, Krebs, Talsma, Woldorff, & Boehler, 2016). Furthermore, proactive inhibitory control can involve adjustments of response-selection thresholds and suppression of motor output to trade speed in the go task for success in the stop task (e.g. Aron, 2011; Verbruggen & Logan, 2009a). The findings of the present study indicate that providing monetary incentives encouraged subjects to make such strategic adjustments^4^ (i.e. subjects in the incentive conditions approached the task differently).

Second, we explored if incentives influenced the dependence between going and stopping on valid-signal trials. Bissett and Logan (2014) found that the ‘no-signal minus signal-respond’ RT difference increased when the proportion of valid signals increased. Thus, the higher proportion of valid signals encouraged the subjects to prioritize stopping (i.e. stopping was less influenced by processing in the primary go task). We expected that incentives on valid-signal trials would have a similar effect. To our surprise, they did not: average signal-respond Go1-RT was shorter than no-signal Go1-RT in the three groups, but there were no statistically significant group by trial type differences (note that this study was sufficiently powered to detect small-effect-sized interactions; see Supplementary Materials). Furthermore, we found that signal-respond Go1-RT was longer than no-signal Go1-RT for a similar subset of subjects in all groups (Figure S1). Finally, we observed similar ‘no-signal vs. invalid-signal’ Go1-RT differences in the three groups. Combined, these findings indicate that signal processing was not influenced by reward or punishment.

It is possible that the high proportion of invalid-signal trials discouraged subjects from prioritizing the stop task on signal trials (Bissett & Logan, 2014). However, this did not discourage them from generally slowing down their Go1 responses, as indicated by the no-signal trial analyses. In other words, our incentive manipulation encouraged subjects to change attentional and/or response settings in the primary go task, but they could not change the level of competition between going and stopping on signal trials. Slowing of all Go1 responses may be the ‘default’ strategy when stopping is incentivized or when subjects expect a signal in the near future (e.g. when a traffic sign informs car drivers that they are near a school or playground, they slow down; they do not wait until they see children crossing the road to adjust their driving). Future proactive inhibitory control studies should further explore which factors influence strategy selection (including the optimality of various response strategies; see e.g. Miller et al., 2009).

### Alternative explanations for the response-slowing

We propose that slowing on no-signal trials reflects proactive control adjustments.

It is unlikely that the Go1-RT group differences reflected increased dual-task demands. After all, accuracy on no-signal trials should also be influenced by dual-task demands. As can be seen in the Appendix, go accuracy was similar for all groups.

The slowing could also be due to the retrieval of stimulus–stop associations. Several studies have indicated that responding on no-signal trials is slowed when stimuli or stimulus features of previous stop trials are repeated (e.g. Bissett & Logan, 2011; Rieger & Gauggel, 1999; Verbruggen et al., 2008; Verbruggen & Logan, 2008b). This stimulus-specific slowing has been attributed to the retrieval of stimulus–stop associations: a go stimulus becomes associated with a ‘stop’ representation on a stop trial; when it is repeated on a following no-signal trial, the stop representation is activated via memory retrieval, and this will suppress the go response or interfere with responding (Verbruggen et al., 2014; Verbruggen & Logan, 2008b). On valid-signal trials, the retrieval of such associations would improve stop performance. Guitart-Massip et al. (2012) demonstrated that associative learning in response-inhibition tasks could be influenced by incentives. Thus, in the incentive conditions, the retrieval of stimulus–stop or signal–stop associations could have had a bigger impact on performance than in the control condition.

As mentioned in the sequential analysis section, we could not examine the contribution of stimulus–signal associations directly. Nevertheless, we think that it is unlikely that incentive-induced changes in associative mechanisms can account for group differences in response-slowing on no-signal trials. Subjects only had to stop and change their response on a very small proportion of the trials (i.e. 8.3% of all trials). Thus, the go stimuli should have become associated with going rather than stopping (hence, Go1-RTs should have decreased throughout the experiment; instead, they increased). It seems also unlikely that altered performance on signal trials was influenced much by incentive-induced changes in memory retrieval or associative learning. The signal mapping changed constantly; consequently, the signal of the previous valid signal was repeated only on a small minority of the signal trials. Furthermore, the signal-respond Go1-RT data are inconsistent with a memory-retrieval account. After all, this account makes the same prediction as the task prioritization account: when the stop response is strongly activated, only the fastest trials can escape inhibition. We already explained above that our data were inconsistent with this idea. Finally, we found that the difference between no-signal and invalid-signal trials decreased throughout the experiment. An associative account predicts the opposite.

Another alternative account for our findings is that the response-slowing is due to a gradual build-up of slowing caused by ‘reactive’ control adjustments after the presentation of a signal (see e.g. Bissett & Logan, 2011). Separating the proactive control account and the ‘build-up’ account is difficult in the present study because the incentive manipulation was block-based. However, it seems unlikely that the slowing is entirely due to post-change-signal adjustments. In the sequential analysis, we found that responding was slowed down after the presentation of an invalid or valid change signal, but this slowing was comparable for the three groups. Thus, a post-change-slowing account cannot explain the group differences observed in the main analyses.

In sum, the group differences cannot easily be explained by a pure memory-retrieval account or a post-change-signal adjustment account. We cannot rule out some minor contribution of associative or memory-retrieval mechanisms and post-change-signal adjustments, but it seems that the slowing on no-signal and signal trials is primarily due to strategy adjustments and competition between decisional processes in the go and stop tasks.

### Effect of incentives on change latencies

The change-RT analysis showed that stop-change performance was better in the reward and punishment groups than in the control group. This improvement could be due to proactive control adjustments (see above). Incentives could also have had a more direct effect on reactive control. Previous work suggests that incentives can increase activity in the reactive inhibitory control network (Boehler et al., 2014). However, our Go1-RT analysis suggests that the decision to stop or not was not influenced much by incentives (i.e. we observed similar differences between no-signal Go1-RTs and signal-respond and invalid-signal Go1-RTs in all three groups). This conclusion is further supported by the SSRT analysis. There were no statistically significant SSRT group differences (and the Bayes factors were inconclusive), but there were large change-RT differences. In stop-change tasks, subjects first stop their Go1 response and then execute the change response on valid-signal trials (Verbruggen et al., 2008). Our findings indicate that monetary incentives did not modulate the stop process much, but they did influence the selection and/or execution of the change response.

The absence of a reliable effect on SSRT is inconsistent with previous studies (e.g. Greenhouse & Wessel, 2013; see also Boehler et al., 2012, 2014). Maybe this is due to the nature of the task, as most other studies have used stop-signal tasks in which only one signal could occur. Furthermore, in our SSRT analysis, we had to exclude many subjects for which the assumptions of the independent race model were violated (and as a consequence of the lower *N*, the study could only detect large between-subject differences).^5^ Consistent with this idea, we found effects of reward in the combined analysis. Therefore, the absence of a statistically significant effect on SSRT in the main experiment should be treated with caution. Note that this does not undermine our main conclusion, namely that incentives in our task encouraged response-slowing but did not influence the dependence between going and stopping.

### Reward and punishment have similar effects on stop-change performance

Previous research suggests that reward and punishment may have distinct effects on learning in response-inhibition tasks. For example, subjects learn cue-go/no-go contingencies faster when correct go responses are rewarded and incorrect no-go responses are punished, than the other way around (Guitart-Masip et al., 2011; 2012). This could be due to a hard-wired link between reward/punishment and go/stop, respectively (Guitart-Masip et al., 2011, 2012; Verbruggen et al., 2014). In the present study, performance in the reward and punishment groups was very similar, and Bayesian analyses provided support for the null hypothesis (see also the Bayesian meta-analysis in Footnote 3). We observed very similar results in two pilot studies in which we observed differences between the control group and the reward and punishment groups, but no differences between the two incentive groups (see Supplementary Materials).

Differences in design could potentially explain the apparent inconsistency between our study and the studies of Guitart-Massip et al. (2011, 2012). In their work, cues presented at the beginning of the trial indicated the combination of the go/no-go requirement and the outcome (reward/punishment). Thus, Guitart-Massip and colleagues used a very direct mapping between action and incentive type. In our study, there were no separate cues at the beginning of a trial, and there was no direct mapping between individual signals (i.e. the chequerboards), stopping, and reward/punishment because the signal rules changed constantly. (Note that we changed the rules because our previous work suggests that stopping and going compete more when the demands on the rule-based system are high; Verbruggen & Logan, 2015). In other words, the mapping was indirect in our study, which could explain why we did not observe a difference between reward and punishment.

It is also possible that we did not observe any differences because the effect of local incentives may depend on global incentives. Previous studies suggest that a match between global incentives (e.g. avoiding losing a bonus or obtaining a bonus) and local incentives (e.g. points deducted for incorrect responses or points awarded for correct responses) encourages flexible behavior, whereas a mismatch encourages behavioral inflexibility (Maddox & Markman, 2010). In our experiments, there was a match between the global and local incentives in both the reward group (subjects had to win a bonus and they could win points on every successful valid-signal trial) and the punishment group (subjects had to avoid losing a £2.5 bonus and they could lose points on every unsuccessful valid-signal trial). This could explain why reward and punishment had a similar effect on flexible stop-change performance. Related to this idea, subjects in the punishment condition started with a bonus, so they could not lose their own money. Consequently, the main task goal could have been similar in both groups, namely trying to maximizing the bonus by accurate performance.

Finally, it could be argued that both conditions involved some reward and punishment. In the punishment condition, subjects were punished for unsuccessful trials, but preventing a loss on successful trials might have been rewarding. In the reward group, subjects received a reward for successful trials, but the absence of a reward on unsuccessful trials could have been perceived as a negative event (see e.g. Verbruggen, Chambers, Lawrence, & McLaren, 2016). Thus, it could be argued that both the punishment and reward groups contained some elements of reward (i.e. getting extra points or avoiding losing points) and punishment (i.e. losing points or not receiving extra points).

The present study cannot distinguish between these various accounts. Therefore, future research is required to test how different reward and punishment schemes can influence performance in the stop task and other cognitive paradigms.

### Conclusions

The present study showed that providing monetary incentives influenced both proactive slowing and reactive control (i.e. execution of a non-dominant, secondary response) in a selective stop-change task. By contrast, task prioritization or the competition between going and stopping after a signal was presented was not influenced much by incentives. Furthermore, we found no effect of the type of (local) incentive.

### Electronic supplementary material

Below is the link to the electronic supplementary material.

Supplementary material 1 (RTF 515 kb)

## Appendix 1

See Tables 6 and 7.

**Table 6:** Overview of Go1 accuracy on no-signal trials and invalid-signal trials: probability of an accurate Go1 response [*p*(correct)] and probability of a missed Go1 response [*p*(miss)] as a function of part (first half or second half of the experiment), group (control, punishment, and reward), and trial type (no-signal vs. invalid-signal)

	*p*(correct)		*p*(miss)	
	*M*	SD	*M*	SD
Part 1				
Control				
No-signal	0.972	0.025	0.011	0.010
Invalid-signal	0.925	0.062	0.030	0.028
Punish				
No-signal	0.976	0.021	0.011	0.013
Invalid-signal	0.934	0.054	0.019	0.026
Reward				
No-signal	0.975	0.021	0.012	0.023
Invalid-signal	0.932	0.054	0.034	0.039
Part 2				
Control				
No-signal	0.972	0.028	0.012	0.014
Invalid-signal	0.956	0.035	0.020	0.024
Punish				
No-signal	0.982	0.019	0.019	0.032
Invalid-signal	0.955	0.046	0.025	0.034
Reward				
No-signal	0.984	0.014	0.013	0.019
Invalid-signal	0.970	0.030	0.034	0.041

Consistent with our previous research (Verbruggen & Logan, 2009a, 2015), we distinguished between incorrect responses (i.e. subjects executed an incorrect response within the response interval) and missed responses (i.e. subjects did not execute any response within the response interval). The probability of a missed go response was generally very low, and, therefore, not further analyzed

**Table 7:** Overview of the analyses of variance

	*df*1	*df*2	SS1	SS2	*F*	*p*	*η*_gen_^2^
No-signal: *p*(correct)							
Group	2	105	0.003	0.081	1.830	0.165	0.027
Part	1	105	0.001	0.020	7.950	**0.006**	0.015
Group by part	2	105	0.001	0.020	1.857	0.161	0.007
Invalid-signal vs. no-signal: *p*(correct)							
Group	2	105	0.006	0.331	0.972	0.382	0.010
Part	1	105	0.033	0.092	37.252	**0.000**	0.053
Trial type	1	105	0.108	0.107	105.463	**0.000**	0.155
Group by part	2	105	0.002	0.092	1.181	0.311	0.004
Group by trial type	2	105	0.001	0.107	0.298	0.743	0.001
Part by trial type	1	105	0.016	0.056	29.827	**0.000**	0.026
Group: part: trial type	2	105	0.001	0.056	1.283	0.282	0.002

Go accuracy was analyzed by means of mixed ANOVAs with group (control, punishment, reward) as a between-subjects factor, and part (first half. vs. second half of the experiment) as within-subjects factor. For the ‘invalid-signal vs. no-signal’ analysis, we also included trial type as a within-subjects factor. *p*’s < 0.05 are in bold

## Appendix 2

See Table 8.

**Table 8:** Overview of Group comparisons for change-RT after exclusion of subjects whose signal-respond RT was longer than their no-signal RT

Comparison	Diff	Lower CI	Upper CI	*df*	*t*	*p*	BF	*g*
Control vs. punish	92	27	156	37	2.887	**0.006**	6.980	0.906
Control vs. reward	86	25	148	40	2.833	**0.007**	6.375	0.862
Punish vs. reward	−5	−54	43	41	0.220	0.827	0.307	0.066

*p*’s < 0.05 after correct for multiple comparisons are in bold

## Footnotes

1 ‘Processing capacity’ can be formalized as a measure of the rate of processing. A process has limited capacity if its rate decreases as more processes enter the race (see e.g. Logan, van Zandt, Verbruggen, & Wagenmakers, 2014). This could be due to competition between stimulus and response representations (Verbruggen & Logan, 2015).

2 Stop-signal and stop-change studies that have used only one signal indicate that simply presenting a signal does not slow RTs (i.e. signal-respond RT is shorter than no-signal RT in most studies).

3 These pilot studies were designed to examine the effect of reward and punishment on reactive control. We found strong effects of incentives on response-slowing in the primary go task, but only weak effects on measures of reactive control. Therefore, we decided to examine the effect of incentives on response-strategy selection and task prioritization in the present study.

4 In previous studies, we fitted the diffusion model or the linear ballistic accumulator model to our data to determine which processing parameters were adjusted when subjects expected a stop signal to occur (Jahfari et al., 2012; Logan et al., 2014; Verbruggen & Logan, 2009a). In this study, we could not fit such a model to the no-signal data of the individual subjects, because the number of trials was relatively low and some subjects did not make any errors (which caused further problems for the fitting).

5 The study was designed to detect within–between subjects interactions (see “Method” section).
