# Supplementary material for: Effects of reward and punishment on the interaction between going and stopping in a selective stop-change task
Source: Psychol Res. 2016 Nov 25;82(2):353–70. doi: 10.1007/s00426-016-0827-5 (PMC5834561; doi:10.1007/s00426-016-0827-5)
Supplement: Supplementary file 1 — Supplementary material 1 (RTF 515 kb) [file 426_2016_827_MOESM1_ESM.rtf]

Supplementary Materials
Additional Results Main Experiment
	Signal-respond vs. no-signal Go1-RTs. As mentioned in the main manuscript, the independent race model does not make any assumptions about whether the executed response on signal-respond trials should 'match' the stimulus (i.e. up for 'U' and down for 'D') or not. Therefore, we included all executed Go1 responses in the analysis reported in the main manuscript. However, we have also repeated the analysis after exclusion of non-matching responses. The results are shown in Tables S1 and S2. As can be seen, inclusion of these trials did not alter the main findings. 

Table S1. Average reaction time for Go1 responses on signal-respond trials (signal-respond Go1-RT) and the difference between signal-respond Go1-RT and no-signal Go1-RT as a function of part (first vs. second half of the experiment) and group (control, punishment, reward). 

Independent variables	Signal-respond Go1-RT	No-signal Go1-RT minus 
signal-respond Go1-RT	
	M	sd	M	sd	
Part 1					
	Control	697	136	41	75	
	Punish	721	151	26	72	
	Reward	740	175	34	78	
Part 2					
	Control	713	171	47	55	
	Punish	774	200	56	61	
	Reward	810	210	59	64	

Table S2: Latencies were analyzed by means of mixed ANOVAs with group (control, punishment, reward) as a between-subjects factor, part (first half. vs. second half of the experiment) and trial type (signal-respond vs. no-signal) as within-subjects factor. p's < .05 are in bold. 

Variable	df1	df2	SS1	SS2	F	p	η2gen	
Group	2	105	369459	12648646	1.533	.221	0.025	
Part	1	105	346527	1185005	30.705	< .001	0.024	
Trial Type	1	105	206691	306227	70.871	< .001	0.014	
Group by Part	2	105	79527	1185005	3.523	.033	0.006	
Group by Trial Type	2	105	449	306227	0.077	.926	0.000	
Part by Trial Type	1	105	11587	177128	6.868	.010	0.001	
Group:Part:Trial Type	2	105	2831	177128	0.839	.435	0.000	
	Individual data. As discussed in the main manuscript, the independent race model predicts that signal-respond Go1-RT should be shorter than no-signal Go1-RT (i.e. signal-respond Go1-RT minus no-signal Go1-RT should be smaller than 0). As can be seen in Figure S1, signal-respond Go1-RT was numerically longer than no-signal Go1-RT for a subset of the subjects in each group (approximately 25-30% of the subjects).

Figure S1. Numerical difference scores for all subjects in the incentive groups for each part. The numbers in the graph indicate the number of subjects per quadrant (unlike previous work, we used the observed numerical values to determine the number of subjects in each group; see Bissett & Logan, 2014, and Verbruggen & Logan, 2015, for a more elaborate discussion).

	Go1-RT and change-RT distributions. The independent race model predicts that signal–respond and no-signal distributions have a common minimum, but later diverge (see Verbruggen & Logan, 2015). The average Go1 distributions, shown in Figure S2, are consistent with this prediction, although it should be noted that violations of the independence assumption are observed for individual subjects (Figure S1). 
	

Figure S2. Percentile averages for signal–respond trials, no-signal trials, and invalid-signal trials for each group. For this graph, we included incorrectly executed go responses – e.g. when subjects pressed the 'up' key instead of the 'down' key.

	The difference between the no-signal and invalid-signal distributions is consistent with our previous research, and suggests dependence between going and stopping (see Verbruggen & Logan, 2015, for a detailed discussion). Note that the no-signal and invalid signal distributions should overlap for percentiles 10-50, but then diverge substantially if subjects stopped all responses when signals occur. Inspection of the figure indicates that even the fastest go responses, which occurred approximately 150–200 ms after the presentation of the signal, were influenced by the presentation of invalid signals. 
	Figure S3 shows the average distributions of change latencies on successful valid-signal trials (i.e. trials on which subjects correctly suppressed the up/down response and responded to the location of the signal instead). As can be seen, the change-RT distribution of the Control group is shifted to the right. In other words, incentives primarily influenced the mean of the change-RT distribution, rather than the shape of it. 

	

Figure S3. Percentile averages for successful valid-change trials.

	
	Bayesian ANOVA. For the RT analyses, we also calculated Bayes factors for all main effects and interaction contrasts in the ANOVA designs (Rouder, Morey, Speckman, Province, 2012). We calculated the Bayes factors with the BayesFactor package in R, using the default prior (Morey & Rouder, 2015). We computed Bayes factors for all possible combinations of fixed factors and interactions, against the null hypothesis that all effects are 0. To reduce the number of model comparisons, interactions were only allowed if all constituent sub-effects were also included (see Morey & Rouder, 2015). 
	The outcome of the Bayesian analyses are presented in Tables S3-6. The models are rank-ordered based on the Bayes factors, and the favored model is on top of the list. Note that 'subject' was included as a factor for all models, but this factor is not added to the model descriptions in the tables to reduce the amount of text. 
	As can be seen, the Bayesian analyses are largely consistent with the ANOVAs reported in the main manuscript. The favored models in Tables S3-S5 included the Part by Group interaction, which provides further support for the idea that RT increased more throughout the experiment in the incentive groups than in the control group. Furthermore, the favored models in Tables S4 and S5 did not include the Group by Trial Type interactions, providing further support for the conclusions that incentives did not influence the difference between no-signal trials and signal trials. In other words, incentives did not influence task prioritization or the degree of parallel processing. They also did not influence sequential effects (Table S6). 


Table S3: No-signal Go1-RT Bayes analysis. 

Model	Bayes factor	Confidence interval	
part + group + part:group	250041	±6.9%	
part 	184675	±0.86%	
part + group	101370	±1.73%	
group	0.51	±0.97%	


Table S4: Signal-respond vs. no-signal Go1-RT Bayes analysis. 

Model	Bayes factor	Confidence interval	
group + part + group:part + trial 	1.07E+17	±5.31%	
group + part + group:part + trial + part:trial 	4.47E+16	±3.63%	
group + part + group:part + trial + group:trial 	5.28E+15	±3.01%	
part + trial 	4.69E+15	±2.36%	
group + part + trial 	2.89E+15	±4.95%	
group + part + group:part + trial + group:trial + part:trial 	2.36E+15	±4.98%	
part + trial + part:trial 	1.99E+15	±4.09%	
group + part + trial + part:trial 	1.23E+15	±4.88%	
group + part + group:part + trial + group:trial + part:trial + group:part:trial 	2.83E+14	±8.13%	
group + part + trial + group:trial 	1.52E+14	±4.15%	
group + part + trial + group:trial + part:trial 	5.54E+13	±2.56%	
group + part + group:part 	8.65E+10	±1.95%	
part 	7.79E+09	±1.95%	
group + part 	4.49E+09	±2.31%	
trial 	5.89E+04	±1.39%	
group + trial 	3.32E+04	±2.99%	
group + trial + group:trial 	1.68E+03	±2.52%	
group 	5.48E-01	±1.61%	
Note: trial = trial type (signal-respond vs. no-signal). 


Table S5: Invalid-signal vs. no-signal Go1-RT Bayes analysis. 

Model	Bayes factor	Confidence interval	
part + trial + part:trial + group + part:group 	9.88E+35	±5.76%	
part + trial + group + part:group 	5.37E+34	±7.27%	
part + trial + part:trial + group + part:group + trial:group 	4.69E+34	±5.23%	
part + trial + part:trial + group + part:group + trial:group + part:trial:group	4.96E+33	±8.8%	
part + trial + part:trial 	4.12E+33	±1.85%	
part + trial + part:trial + group 	2.43E+33	±6.36%	
part + trial + group + part:group + trial:group 	2.20E+33	±3.59%	
part + trial 	2.78E+32	±4.85%	
part + trial + group 	1.75E+32	±9.57%	
part + trial + part:trial + group + trial:group 	1.15E+32	±4.6%	
part + trial + group + trial:group 	7.39E+30	±6.24%	
trial 	5.64E+24	±0.75%	
trial + group 	3.50E+24	±3.1%	
trial + group + trial:group 	1.57E+23	±4.19%	
part + group + part:group 	5.82E+05	±3.47%	
part 	7.14E+04	±0.81%	
part + group 	6.65E+04	±42.85%	
group 	5.32E-01	±1.23%	
Note: trial = trial type (invalid-signal vs. no-signal). 

Table S6: Sequential no-signal Go1-RT Bayes analysis. 

Model	Bayes factor	Confidence interval	
previous trial	1.26E+18	±0.59%	
previous trial + group 	7.95E+16	±0.92%	
previous trial + group + previous trial:group	7.41E+15	±1.11%	
group	0.054	±0.53%	
Note: previous trial = a correct no-signal trial, a correct invalid-signal trial, a successful (signal-inhibit) valid-signal trial, or an unsuccessful (signal-respond) valid-signal trial.

Two Pilot Experiments 
	In two pilot behavioral experiments, we examined the effects of reward and punishment on performance in two other variants of the stop-signal task. The tasks were optimized for ERP purposes (initially we were planning a series of ERP studies to examine the effects of reward and punishment on 'reactive' attentional and response-related processes on signal trials). Note that sensitivity analyses showed that these experiments could only detect large between-group differences. 
	In both experiments, there were three groups: a punishment group, a reward group, and a control group. In Pilot Experiment 1 (N = 72; 24 subjects per group), we used a hybrid version of a go/no-go task and a stop-change task (as used in Elchlepp & Verbruggen, 2016); in Pilot Experiment 2, we used a stop-signal task (N = 108; 36 subjects per group). All signals were valid and we used a very strict response-deadline procedure to discourage proactive slowing. 
	A detailed overview of the experiments, methods, results, and all raw data and analysis scripts are deposited in the Open Research Exeter data repository (http://hdl.handle.net/10871/18924). In both experiments, punishment and reward influenced measures of reactive and proactive control (despite the strict response deadline on no-signal trials). Importantly, Bayesian analyses indicated that there were no differences between the Reward and Punishment groups, which is consistent with the results reported in the main manuscript. 
	
References
Elchlepp, H., & Verbruggen, F. (2016). How to withhold or replace a prepotent response:  An analysis of the underlying control processes and their temporal dynamics. Biological Psychology, accepted pending minor revisions 
Richard D. Morey and Jeffrey N. Rouder (2015). BayesFactor: Computation of Bayes Factors for Common Designs. R package version 0.9.12-2., https://CRAN.R-project.org/package=BayesFactor
Rouder, J. N., & Morey, R. D. (2011). A Bayes factor meta-analysis of Bem's ESP claim. Psychonomic Bulletin & Review, 18, 682–689. http://doi.org/10.3758/s13423-011-0088-7
Rouder, J. N., Morey, R. D., Speckman, P. L., & Province, J. M. (2012) Default Bayes Factors for ANOVA Designs. Journal of Mathematical Psychology, 56, 356-374.
Verbruggen, F., & Logan, G. D. (2015). Evidence for capacity sharing when stopping. Cognition, 142, 81–95. http://doi.org/10.1016/j.cognition.2015.05.014
